# Design and Validation of a Scale of Knowledge of Cardiovascular Risk Factors and Lifestyle after Coronary Event

**DOI:** 10.3390/jcm11102773

**Published:** 2022-05-14

**Authors:** María Ángeles Bernal-Jiménez, Germán Calle-Pérez, Alejandro Gutiérrez-Barrios, Livia Gheorghe, Ana María Solano-Mulero, Nuria Trujillo-Garrido, Amelia Rodríguez-Martín, Josep A. Tur, Rafael Vázquez-García, María José Santi-Cano

**Affiliations:** 1 Faculty of Nursing and Physiotherapy, University of Cádiz, 11003 Cádiz, Spain; mariangeles.bernal@uca.es (M.Á.B.-J.); nuria.trujillo@uca.es (N.T.-G.); amelia.rodriguez@uca.es (A.R.-M.); 2 Institute of Biomedical Research and Innovation of Cádiz (INiBICA), 11009 Cádiz, Spain; gcallep@hotmail.com (G.C.-P.); aleklos@hotmail.com (A.G.-B.); livia_gheorghe_ro@yahoo.es (L.G.); rafael.vazquez.sspa@juntadeandalucia.es (R.V.-G.); 3 Research Group on Nutrition: Molecular, Pathophysiological and Social Issues, University of Cádiz, 11003 Cádiz, Spain; 4 Cardiology Unit, Puerta del Mar Hospital, 11009 Cádiz, Spain; anamasom@gmail.com; 5 Biomedicine, Biotechnology and Public Health Department, University of Cádiz, 11003 Cádiz, Spain; 6 Research Group on Community Nutrition & Oxidative Stress, University of the Balearic Islands-IUNICS, IDISBA & CIBEROBN, 07122 Palma de Mallorca, Spain; pep.tur@uib.es

**Keywords:** scale, knowledge, cardiovascular risk factors, lifestyle, coronary disease

## Abstract

Background: It is important for health professionals to have tools available to assess patients’ knowledge of lifestyle and cardiovascular risk factors after they have suffered a coronary event and determine whether educational interventions are effective. This study aims to design and validate a scale to evaluate this knowledge. Methods: Four-phase instrument design: (A) Conceptual review. (B) Review by experts. (C) Pilot test–retest. (D) Psychometric validation of the final version of the questionnaire with 24 items. A panel of experts performed the content validity. The reliability of the scale was measured using Cronbach’s alpha score and criterion validity was evaluated by comparing the total scores for knowledge obtained by the participants among the three education level groups. The construct and dimensional structure validity were assessed using exploratory factor analysis. Results: A total of 143 people participated, 30 in the pilot study and 113 (68% male, 60.2 ± 9 years) in the psychometric validation of version 3 of the scale. A Cronbach’s alpha score of 0.887 was reached for this version. The factor analysis showed that the items were distributed into five factors that explained 57% of the variance. Significant differences were observed in the level of knowledge among the patients of the three levels of education (low, moderate and high) (99.20 ± 11.93, 105.92 ± 7.85, 109.78 ± 8.76 points, *p* = 0.003), as there was a negative correlation between age and knowledge level (r = −0.213, *p* = 0.024). Conclusions: The scale presents psychometric properties that are evidence of its reliability and validity. The relationship demonstrated between the level of knowledge and age, sex and level of education shows the importance of emphasizing educational interventions for elderly people and those with a lower level of education.

## 1. Introduction

Coronary heart disease (CHD) is one of the main causes of morbimortality around the world. Ischemic heart disease is responsible for 16% of the total deaths from cardiovascular disease (CVD) [1]. In addition, the prevalence of unhealthy lifestyle habits is high [2], which highlights the importance of both primary and secondary prevention strategies to prevent or decrease recurrence and mortality after suffering a coronary event [3,4].

Greater knowledge of CVD and its risk factors, together with a healthy lifestyle, is shown to reduce the risk of suffering from it and encourages appropriate healthcare decisions to be taken [3,5,6]. A study performed among patients with CHD after stent implantation reported deficient knowledge of their disease. The authors concluded that it was necessary to implement innovative strategies to increase their knowledge and provoke lifestyle changes [7]. Clinical practice guidelines for preventing CVD recommend developing cardiac rehabilitation programs beginning before hospital discharge to improve patients’ knowledge [3]. However, the information on the best methods to assess patient knowledge prior to the beginning of such a program is limited.

Several questionnaires testing the knowledge of CVD among the general population and patients with CVD have been developed and validated [8,9,10]. However, these focused more on the symptoms of the disease itself and awareness of the risk of suffering cardiovascular events than on knowledge and attitudes towards modifiable cardiovascular risk factors (CVRFs) and lifestyle in people that have suffered from a cardiac event.

Therefore, it is important that health professionals have tools to assess patients’ initial understanding of cardiovascular risk factors and lifestyle and check whether educational interventions are effective.

The main objective of this study was to design and validate a scale to assess the level of knowledge and attitude towards modifiable CVRFs and the recommended lifestyle for people with CHD. The secondary objectives were to assess the relationship between the level of knowledge, age, sex, education level, and history of CVRFs.

## 2. Materials and Methods

### 2.1. Study Design

**Figure 1:**
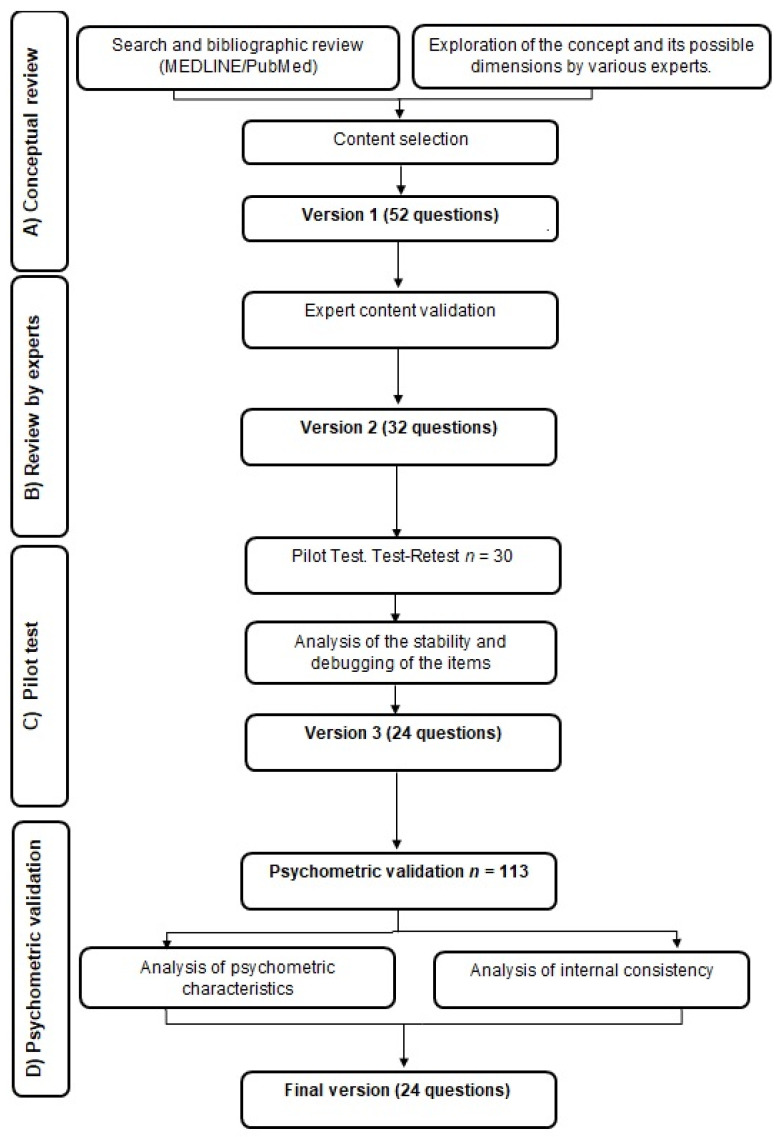
General schema of the development of the study to design and validate a scale to assess knowledge of cardiovascular risk factors and the lifestyle recommended after a coronary event.

An observational prospective study for the design and validation of a self-administered scale to assess the level of knowledge of modifiable CVRFs among patients that have suffered from a coronary event with stent implantation attended in the Cardiology Unit of a public specialty reference hospital in the province of Cádiz, Spain, was developed. The study consisted of four phases (Figure 1).

### 2.2. Conceptual Review

#### Design of the Scale

**Figure 2:**
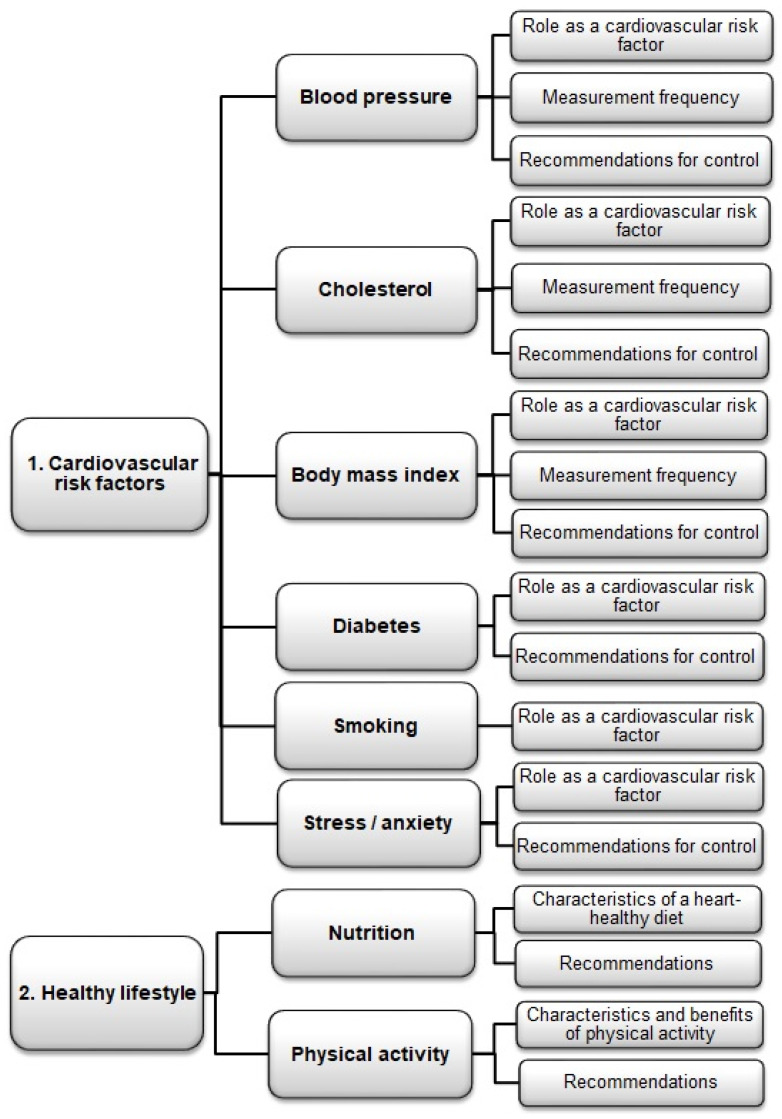
Contents identified and selected in search of the literature into knowledge and attitudes about cardiovascular risk factors and the lifestyle recommended for managing them after a coronary event.

To identify the most important information that patients with CHD need to know about CVRFs and their control through a healthy lifestyle, a conceptual review was performed with a search in the MEDLINE/PubMed database between March and May 2019, later updated to September 2021 [3,11,12,13]. Furthermore, a review of the literature was performed on validated instruments that assess knowledge of the control of CVRFs and the lifestyle recommended for people with CHD. The search terms were as follows: coronary artery disease, cardiovascular disease, heart disease, cardiovascular risk factor, lifestyle, guidelines, blood pressure, hypertension, diabetes mellitus, dyslipidemias, cholesterol, obesity, body mass index, smoking, stress, anxiety, physical activity, nutrition, secondary prevention, patient education, health, knowledge, attitudes, behavior, questionnaires and psychometric validation, combining them using the Boolean operators (AND/NOT) and filtering the appearance of these terms into Title/Abstract. This review highlighted a lack of validated instruments specifically for this issue. The published questionnaires focused mainly on the knowledge of CVD itself, its symptoms and awareness of the risk of suffering from it [8,9,10]. With this information, an exploration was begun of the concept of “knowledge and lifestyle” in people with CHD. This was performed by two researchers, two nurses and two cardiologists with experience in these factors and their respective specialties (Figure 2). A scale (version 1) was designed with 52 positive and negative items in similar proportions on a Likert-type scale (Score 1 to 5: completely agree, agree, not sure, disagree and completely disagree, the highest score given to the most correct response).

### 2.3. Review by Experts

#### Validation of Content by Experts

Between June and September 2019, consultation took place with a group of five experts: a nurse, a specialist doctor in endocrinology and three specialist cardiologists. The experts gave their opinions and/or suggestions regarding modifications to the instrument.

The changes proposed were modifications to how some of the items were written and the elimination of others, validating the content of the scale and obtaining version 2 with 32 items.

### 2.4. Pilot Test: Test–Retest

In October 2019, the pilot test was conducted through two self-administered applications (test–retest) of version 2 of the scale (32 items) involving patients with CVD attended in the cardiology department. Five days elapsed between the test and retest (Table 1).

#### 2.4.1. Stability Analysis

Pearson’s correlation coefficient was calculated for the two applications of version 2 of the instrument, considering values of 0.70 to be indicative of the instrument being stable.

#### 2.4.2. Applicability of the Instrument and Refining the Items

The results of the test were analyzed according to the following criteria: level of complexity of the questions (percentage of correct and incorrect answers), the correlation between the score obtained in each item and the global score (item/total correlation), internal consistency and the time required to complete the scale.

Analyzing the results allowed the group of experts to reach a consensus with the researchers about the modifications that would lead to version 3 of the scale with 24 items. The general characteristics of the patients (Table 2) and the content of version 3 are shown in Table 3.

### 2.5. Psychometric Validation

The psychometric validation of the test was conducted between November 2019 and March 2021.

#### 2.5.1. Participants

Patients were recruited in the EVITE clinical trial for educational intervention after a coronary event [14].

##### Inclusion Criteria

Adults over 18 and under 75 years of age who had suffered from a coronary event with first stent implantation at the time of the study.

##### Exclusion Criteria

Patients with severe heart failure, physical disability or dementia, serious congenital, structural or rheumatic heart disease or chronic liver or kidney disease.

#### 2.5.2. Sample Size

A sample size of 108 people was estimated for a confidence level of 95%, a proportion of patients with acceptable knowledge of 50% [15], an accuracy of 5% and a population attended in the unit fulfilled the inclusion criteria during the study period of 150 patients. This was based on Hair and Anderson’s recommendation of a sample of at least 100 participants [16]. Finally, version 3 of the scale (24 items) was administered prospectively to 113 patients with CHD.

#### 2.5.3. Psychometric Characteristics

The internal consistency of the scale was assessed by calculating the item-total correlation coefficient to check the degree of correlation between each variable and the total score, values equal to or above 0.200 being considered valid. Cronbach’s alpha index score was also calculated for the set of items and their dimensions. For this index, values of 0.6 are considered acceptable, and those equal to or above 0.7 are good [17]. The degree of feasibility was determined from the percentage of answers obtained and the time taken to complete the scale.

Construct validity was analyzed by exploratory factor analysis, considering loading coefficients greater than 0.4. This procedure culminated in version 3 being approved (definitive version).

Regarding evaluating the level of knowledge, the maximum score on the scale is 120 points since it consists of 24 items with five response options scoring from 1 to 5 points, the most correct option scoring 5 points. Subjects were considered to have a high level of knowledge when they chose the correct response for over 75% of the items (90 points) [15].

The criterion validity was evaluated by comparing the total scores for knowledge obtained by the participants among the three education level groups: primary education (low), secondary education (medium) and university education (high) [18].

### 2.6. Clinical Variables Studied

An analysis was performed of the following variables: sociodemographic variables (age, sex, education level), history of cardiovascular risk factors (diabetes mellitus, high blood pressure, dyslipidemia, obesity, tobacco use, the total number of CVRF and cardiovascular risk before the coronary event expressed as a percentage (low-level risk <5%, moderate 5–9.9%, high 10–14.9%, and very high ≥15%) obtained using the online calculator of the Regicor study (https://regicor.cat/es/aplicaciones/regicor/, accessed on 15 September 2021) [19], personal and family history of CVD, plasma analysis (total cholesterol, LDL cholesterol, HDL cholesterol) and treatment prescribed before the event. In addition, anthropometric variables (weight, height, body mass index and waist circumference), blood pressure and heart rate were measured.

### 2.7. Statistical Analysis of the Data

The data obtained during the process were analyzed with version 24.0 of the SPSS software for Windows (IBM, Armonk, NY, USA). The descriptive statistics are presented as absolute and percentage frequencies for the qualitative variables and as dispersion measurements (standard deviation) and central tendency (mean) for the continuous variables.

In addition to the psychometric parameters required for the validation (Cronbach’s alpha and Pearson correlation coefficient), a descriptive analysis was performed of the baseline characteristics of the participants and a test was conducted on the relationship of the scores with sex, using student’s *t* statistics for independent samples, and with the education level using an ANOVA for more than two independent samples and the Pearson correlation coefficient. The Kaiser–Meyer–Olkin measure of sampling adequacy and Bartlett’s test of sphericity were previously performed. Next, exploratory factor analysis was performed using principal component extraction and varimax rotation, including all of the items. Statistical significance was set at a 95% confidence level.

### 2.8. Ethical and Legal Aspects

The study was conducted in agreement with the guidelines and protocols established in the Helsinki Declaration as revised in Fortaleza (Brazil) in October 2013 and complies with Law 14/2007 on Biomedical Research and with European Data Protection Regulations. It was approved by the Biomedical Research Ethics Committee of the Costa del Sol, Andalusia, with the reference: 003_ene19_PI-EVITE-18.

All the participants were informed that their answers would be analyzed as part of a research study. Anonymity and the correct treatment of personal data were guaranteed. All participants signed informed consent forms, and the privacy and confidentiality of the data included were ensured.

## 3. Results

A total of 143 patients participated in the pilot study and the psychometric validation.

### 3.1. Pilot Test

The pilot test was performed with 30 participants. Version 2 of the scale with 32 questions was used. The internal consistency and stability of the test were analyzed. The Pearson correlation coefficient values in the pilot test–retest generally showed that the instrument presented good stability throughout the process. The Cronbach’s alpha score (Test: a = 0.695; retest: a = 0.756) indicated that the scale had acceptable internal consistency. Despite most items reaching an item-total correlation above 0.200, some presented a lower correlation and were eliminated (Table 1).

**Table 1:** Cronbach’s alpha coefficient, reliability and stability of version 2 (32 questions) in the pilot test–retest.

		Test	Retest
	Cronbach’s Alpha	0.695	0.756
	Reliability/Internal Consistency		Stability
Questions	Item-Total Correlation		Pearson Correlation Coefficient
	Test	Retest	Test–Retest
Q 1	0.458	0.450	0.537
Q 2	0.201	0.236	0.545
Q 3	−0.113	0.260	0.406
Q 4	0.419	0.273	0.580
Q 5	0.331	0.126	0.447
Q 6	0.076	0.069	0.734
Q 7	0.519	0.402	0.447
Q 8	0.194	0.344	0.659
Q 9	0.530	0.195	0.272
Q 10	0.301	0.456	0.547
Q 11	0.504	0.561	0.660
Q 12	0.516	0.508	0.705
Q 13	−0.147	−0.039	0.324
Q 14	0.348	0.243	0.605
Q 15	0.320	0.657	0.403
Q 16	0.216	0.171	0.076
Q 17	0.435	0.321	0.405
Q 18	0.509	0.381	0.663
Q 19	0.031	0.345	0.227
Q 20	0.187	0.429	0.267
Q 21	0.582	0.576	0.459
Q 22	0.268	0.374	0.454
Q 23	0.556	0.411	0.557
Q 24	0.365	0.588	0.447
Q 25	0.070	0.565	0.407
Q 26	0.357	0.166	0.465
Q 27	0.357	0.553	0.639
Q 28	0.351	0.530	0.715
Q 29	0.325	0.319	0.501
Q 30	0.642	0.597	0.662
Q 31	0.405	0.289	0.245
Q 32	0.088	0.419	0.131

Q1. I consider that only adults with high blood pressure should measure their blood pressure regularly. Q2. High blood pressure predisposes to heart disease. Q3. Blood pressure of 150/90 mmHg (or 15/9) is high. Q4. Diet and doing physical exercise help to lower blood pressure. Q5. It is recommended that adults have regular tests to monitor blood cholesterol. Q6. A blood cholesterol level greater than 175 mg/dL is high. Q7. Following a diet and doing physical exercise is not a very effective way to lower blood cholesterol levels. Q8. High blood cholesterol influences the onset of cardiovascular disease. Q9. Bodyweight has little influence on cardiovascular health. Q10. Increased fat around the waist increases the risk of cardiovascular disease and diabetes. Q11. I consider that it is NOT necessary to measure body weight regularly. Q12. Overweight and obesity hardly increase the risk of cardiovascular disease. Q13. A person can have diabetes without showing symptoms. Q14. People with diabetes treated with pills or insulin should follow a balanced diet. Q15. High blood sugar hardly increases the risk of cardiovascular disease. Q16. Tobacco is harmful to cardiovascular health. Q17. Being a passive smoker hardly increases the risk of suffering from cardiovascular disease. Q18. Being a light smoker is NOT harmful to your health. Q19. Stress increases cardiovascular risk. Q20. Reducing stress improves cardiovascular health. Q21. Stress hardly influences cardiovascular health. Q22. Doing exercise reduces stress. Q23. Food has little influence on cardiovascular health. Q24. It is better to eat fresh food than ready-made food. Q25. Fish should be eaten every week. Q26. Red meat (beef, pork) should be eaten infrequently. Q27. Vegetables should be eaten every day. Q28. Fruits should be eaten every day. Q29. People should walk for 30–45 min every day. Q30. Doing physical activity hardly improves cardiovascular health. Q31. Lack of physical exercise makes you more likely to suffer from cardiovascular diseases. Q32. Physical activity improves mood.

### 3.2. Psychometric Validation

A total of 113 patients participated, of whom 77 were men (68.1%), with a mean age of 60.25 ± 9.04 (range 38–75). The general characteristics of the participants are shown in Table 2. Among the participants, 99.5% answered all the questions. The mean time taken was 15 min.

**Table 2:** General characteristics of the participants in the psychometric validation *n* = 113.

**Gender % (*n*)**	
Men % (*n*)	68.1 (77)
Women % (*n*)	31.9 (36)
**Age mean ± SD**	60.25 ± 9.04
Men mean ± SD	58.48 ± 9.18
Women mean ± SD	64.03 ± 7.57
**Obesity % (*n*)**	42.5 (48)
BMI (kg/m^2^) mean ± SD	29.28 ± 4.94
Waist circumference (cm) mean ± SD	104.57 ± 10.52
**HTN % (*n*)**	57.5 (65)
SBP (mmHg) mean ± SD	132.12 ± 19.26
DBP (mmHg) mean ± SD	75.24 ± 10.57
Heart rate (beats/minute) mean ± SD	73.78 ± 12.44
**Diabetes % (*n*)**	33.6 (38)
**Dyslipidemia % (*n*)**	53.1 (60)
Total Cholesterol (mg/dL) mean ± DE	188.03 ± 51.47
LDLc (mg/dL) mean ± DE	121.35 ± 43.12
HDLc (mg/dL) mean ± DE	41.51 ± 12.16
**Smokers % (*n*)**	37.2 (42)
**Former smoker % (*n*)**	31.9 (36)
**Number of cardiovascular risk factors mean ± SD**	2.22 ± 1.15
**Cardiovascular risk (%) mean ± SD**	6.82 ± 3.43
**Personal history CVD % (*n*)**	6.2 (7)
NSTEMI % (*n*)	0.9 (1)
STEMI % (*n*)	3.5 (4)
Stable angina % (*n*)	1.8 (2)
**Family history**	
Angina % (*n*)	4.4 (5)
Heart attack % (*n*)	27.4 (31)
**Current reason PCI**	
Stable angina % (*n*)	27.4 (31)
Unstable angina % (*n*)	17.7 (20)
NSTEMI % (*n*)	21.2 (24)
STEMI % (*n*)	33.6 (38)
**Previous Treatment**	
Anticoagulants % (*n*)	1.8 (2)
Antiplatelet % (*n*)	31.0 (35)
B-blockers % (*n*)	22.1 (25)
Calcium channel blockers % (*n*)	17.7 (20)
ACE-I % (*n*)	21.2 (24)
ARB II % (*n*)	28.3 (32)
Nitrates % (*n*)	7.1 (8)
Diuretics % (*n*)	19.5 (22)
Insulin % (*n*)	8.8 (10)
Oral Antidiabetics % (*n*)	27.4 (31)
Estatins % (*n*)	46.0 (52)
Omeprazole % (*n*)	35.4 (40)
Other % (*n*)	46.0 (52)

ACE-I: angiotensin-converting enzyme inhibitors; ARB II: Angiotensin II receptor antagonists; BMI: body mass index; CVD: cardiovascular diseases; DBP: diastolic blood pressure; HDLc: High-density lipoprotein cholesterol; LDLc: Low-density lipoprotein cholesterol; NSTEMI: Non-ST-elevation myocardial infarction; SBP: systolic blood pressure; STEMI: ST-elevated myocardial infarction.

The analysis of the reliability of version 3 (24 items) is shown in Table 3. 

**Table 3:** Cronbach’s alpha coefficient and reliability of Version 3 (24 items) in the psychometric validation.

Questions		
	Cronbach’s Alpha	0.887
		Reliability Internal Consistency.
		Item-Total Correlation
Q 1	I consider that only adults with high blood pressure should measure their blood pressure regularly.	0.410
Q 2	High blood pressure increases the risk of cardiovascular disease.	0.485
Q 3	Diet and doing physical exercise help to lower blood pressure.	0.546
Q 4	All adults should have a regular blood test to monitor blood cholesterol levels.	0.582
Q 5	Following a diet and doing physical exercise is not a very effective way to lower blood cholesterol levels.	0.687
Q 6	Having high blood cholesterol levels increases the chances of suffering from cardiovascular diseases.	0.567
Q 7	Bodyweight has little influence on cardiovascular health.	0.665
Q 8	I consider that it is NOT necessary to measure body weight regularly.	0.518
Q 9	Overweight and obesity increase the risk of cardiovascular disease.	0.566
Q 10	Weight loss in obese people helps to control diabetes.	0.470
Q 11	Diet is a part of the treatment of diabetes.	0.569
Q 12	High blood sugar hardly increases the risk of cardiovascular disease.	0.548
Q 13	Tobacco is harmful to cardiovascular health.	0.391
Q 14	Being a passive smoker hardly increases the risk of suffering from cardiovascular disease.	0.399
Q 15	Being a light smoker is NOT harmful to your health.	0.433
Q 16	Stress hardly influences cardiovascular health.	0.728
Q 17	Doing exercise reduces stress.	0.643
Q 18	Stress is harmful to cardiovascular health.	0.541
Q 19	Food has little influence on cardiovascular health.	0.512
Q 20	It is better to eat fresh food than ready-made food.	0.508
Q 21	Eating fruit and vegetables every day is recommended.	0.469
Q 22	People should walk for 30–45 min every day.	0.409
Q 23	Doing physical activity hardly improves cardiovascular health.	0.718
Q 24	Lack of physical exercise makes you more likely to suffer from cardiovascular diseases.	0.563

With this version, a Cronbach’s alpha score of 0.887 was reached, considered to be good–excellent. 

The Kaiser–Meyer–Olkin measure of sampling adequacy and Bartlett’s test of sphericity (0.613) suggested the possibility of conducting the factor analysis to check the construct validity. The factor analysis of the main components showed that the items were distributed into five factors that explained 57% of the variance (Table 4).

The Cronbach’s alpha score was calculated for each dimension, as were the correlations of each item with its dimension (Table 4). The scores were as follows: dimension 1 (0.821), dimension 2 (0.829), dimension 3 (0.725) and dimension 4 (0.505).

The mean total score on the scale was 104.7 ± 9.7 points. A score of over 90 points was obtained by 91.2% of the patients, meaning that they gave the correct responses to at least 75% of the items.

A negative correlation was observed between age and the level of knowledge (r = −0.213; *p* = 0.024), which indicates that the older participants knew less. Regarding gender, the men had a significantly higher mean score in the level of knowledge (105.9 ± 9.5429) than the women (102.96 ± 9.772) (*p* = 0.046) (Table 5).

The ANOVA test showed significant differences (*p* = 0.003) in the level of knowledge according to education level. The participants with a higher level of education obtained a mean score of 109.78 ± 8.762, those with a medium level had a mean score of 105.92 ± 7.851, and those with a primary education obtained a mean score of 99.20 ± 8.762 (Table 5).

## 4. Discussion

The present study designed and validated an instrument for measuring the level of knowledge of CVRFs and the recommended lifestyle for controlling these factors in people with CHD. The scale demonstrated its reliability and validity through an internal consistency (Cronbach’s alpha) of 0.887, considered to be good–excellent [17], a content validity verified with experts, construct validity through factor analysis, criterion validity backed by the relationship between the level of knowledge and education level and expected variables such as age or sex. Furthermore, it is a self-administered scale that can be conducted in 15 min and was completed by practically all the participants.

To our knowledge, there are currently no validated questionnaires testing the knowledge of CVRFs and healthy lifestyles in people that have suffered from a coronary event. In a study conducted in Sweden as part of the EUROASPIRE II study in patients that had experienced a coronary event 6 months earlier and had attended a cardiac rehabilitation program, a 28-item questionnaire was designed to determine whether knowledge of CVRFs was related to changes in lifestyle and adherence to treatment. The questionnaire included questions about general knowledge of CVRFs, specific knowledge of whether they thought that these factors affected their CHD, their prescribed treatment and their adherence to it [20]. This questionnaire was later validated [21], obtaining reliability, measured by a Cronbach’s alpha score of 0.73, although the authors did not analyze the instrument’s construct validity. Neither did this study establish a global score for the questionnaire nor cut-off points for levels of knowledge. The authors concluded that a correlation existed between specific knowledge of coronary heart disease itself and self-reported changes in lifestyle and treatment compliance.

In a study performed in Spain, the authors validated an instrument that measured the level of knowledge of the risk of CVD in patients taking cardiovascular medication and going to community pharmacies. In the validation, the questionnaire showed a Cronbach’s alpha score of 0.88, and its reliability and external validity were also tested. Nevertheless, this questionnaire contained items oriented towards knowledge of CVD and the risk of suffering a myocardial infarction rather than CVRFs and a healthy lifestyle specifically. Moreover, patients that had had an acute myocardial infarction were excluded.

In our study, the dimensional structure of the scale and its construct validity were evaluated using exploratory factor analysis, which identified five factors that explained 57% of the variance. The first three factors included five items each, while the fourth grouped four items and the fifth three items. Although item 8 remained the only component in factor 6, and item 7 was not shown in the component matrix, the decision was made to keep both items on the scale as they referred to the effect of body weight on cardiovascular health and the importance of measuring it regularly. It was considered that both contents should be included in educational prevention strategies programs for CVD and should therefore be assessed.

To measure levels of knowledge, appropriate instruments are required, such as reliable, validated questionnaires [8,9,10]. However, there is no gold standard for measuring the level of knowledge of CVRFs, resulting in many of the studies performed over the years having created questionnaires to this end [21,22,23,24]. Most of these were designed to assess knowledge of CHD itself and awareness of the risk of suffering from CVD but did not specifically focus on the role that CVRFs play in the development of CHD.

Knowledge of the disease and its risk factors is considered a prerequisite for making decisions about health care [24]. This knowledge helps patients control risk factors and adopt behavior that promotes cardiovascular health [24,25]. Moreover, people must be motivated and willing to participate actively in adopting a healthy lifestyle [25,26].

Regarding the level of knowledge, over 90% of the patients in the present study answered 75% or more of the items correctly (≥90 points). This suggests that patients had a high level of knowledge, possibly due to them having a history of CVRFs under treatment and for which they had received advice. These results are in line with those from the CADE-II study performed on patients with CHD in a cardiac rehabilitation program, in which a high initial level of knowledge was also observed [15]. In another study, patients with CHD also had a high level of knowledge of cardiovascular disease [27].

A negative correlation was observed between the level of knowledge and age; in other words, the older participants received lower scores on the knowledge scale than the younger ones. These data coincide with the results found by several other studies [23,28,29,30]. However, another study conducted on patients with cardiometabolic risk factors did not report this relationship [29].

We also found that the men scored significantly higher on the scale than the women, unlike in other studies where no differences were observed [27]. Although the results in our study could be attributed to a lower education level connected with gender, age might also be a factor since women usually suffer from coronary events at an older age, as Table 2 show.

Regarding the relationship between the patients’ knowledge and education level, our results show that those with a low education level received a lower score on the scale, as observed in other studies [27,28,29,30]. However, these differences were not found in the CADE-II study, which could be attributed to patients with a low educational level not being represented in the sample. On the other hand, people with a history of heart diseases such as heart failure, cardiomyopathy or percutaneous coronary intervention were shown to present a higher level of knowledge, which could be indicative of having received prior education [15]. By contrast, for most of the participants in our study, the first presentation of CHD was at the time of the study, and no relationship was found between their level of knowledge and the presence of CVRFs.

These results highlight the importance of evaluating the prior knowledge of patients to be able to structure their learning in educational programs and dedicate the necessary time to filling in the gaps in their knowledge. It would also seem to be important to adapt these teaching programs to the particular kind of participants and implement strategies that enable all patients to have access to them, especially those with a lower level of education that are in greater need of acquiring the information provided.

This study presents some limitations. First, the scale was used with patients with CHD and stent implantation, so it would be necessary to extend its application to patients with CHD that receive another kind of treatment such as coronary bypass surgery. Second, criterion validity was assessed in relation to the level of education, and it would be useful to include other validated questionnaires testing knowledge. Third, our study did not take into consideration the patients’ previous learning of CVRFs and lifestyle. Fourth, we designed a Likert-type scale (completely agree, agree, not sure, disagree and completely disagree), and there could be patients who did not understand which corresponded with the correct answer. Fifth, the study took place in a hospital, although this is a reference center that attends to patients from very diverse urban, rural and socioeconomic backgrounds.

Finally, further studies are required to evaluate whether the scale is sensitive to change in such a way that an increase can be observed in the patient’s level of knowledge after educational interventions.

## 5. Conclusions

The scale designed presents psychometric properties that are proof of its reliability and validity for measuring the level of knowledge of CVRFs and the lifestyle recommended for their control among people with CHD. The relationship demonstrated between the level of knowledge and age, sex and education level shows the importance of emphasizing educational interventions for elderly people and those with a lower education level. This tool could be used to analyze the level of knowledge of patients in both primary and secondary prevention. It could also help to assess the effectiveness of an educational intervention aimed at improving knowledge of the control of CVRFs and its relationship with achieving treatment goals and decreasing the onset of future coronary events.

## Figures and Tables

**Table 4:** Variance. Cronbach’s alpha score for each dimension, and item-dimension correlation.

		Variance (%)	Cronbach’s Alpha—Dimension	Dimension—Total Correlation	Item—Dimension Correlation
**Factor 1** Knowledge of lifestyle habits		14.695	0.821	0.844	
Q 16.	Stress hardly influences cardiovascular health.				0.866
Q 17.	Doing exercise reduces stress.				0.735
Q 19.	Food has little influence on cardiovascular health.				0.703
Q 23.	Doing physical activity hardly improves cardiovascular health.				0.835
Q 24.	Lack of physical exercise makes you more likely to suffer from cardiovascular diseases.				0.709
**Factor 2** Knowledge of control of cholesterol and blood pressure		12.916	0.829	0.746	
Q 2.	High blood pressure increases the risk of cardiovascular disease.				0.760
Q 3.	Diet and doing physical exercise help to lower blood pressure.				0.755
Q 4.	All adults should have a regular blood test to monitor blood cholesterol levels.				0.844
Q 5.	Following a diet and doing physical exercise is not a very effective way to lower blood cholesterol levels.				0.703
Q 6.	Having high blood cholesterol levels increases the chances of suffering from cardiovascular diseases.				0.864
**Factor 3** Knowledge of lifestyle recommendations		12.441	0.725	0.660	
Q 13.	Tobacco is harmful to cardiovascular health.				0.516
Q 18.	Stress is harmful to cardiovascular health.				0.749
Q 20.	It is better to eat fresh food than ready-made food.				0.803
Q 21.	Eating fruit and vegetables every day is recommended.				0.814
Q 22.	People should walk for 30–45 min every day.				0.672
**Factor 4** Knowledge of cardiovascular risks		8.650	0.505	0.582	
Q 1.	I consider that only adults with high blood pressure should measure their blood pressure regularly.				0.657
Q 9.	Overweight and obesity increase the risk of cardiovascular disease.				
Q 14.	Being a passive smoker hardly increases the risk of suffering from cardiovascular disease.				0.759
Q 15.	Being a light smoker is NOT harmful to your health.				0.729
**Factor 5** Knowledge of diabetes mellitus		8.340	0.696	0.683	
Q 10.	Weight loss in obese people helps to control diabetes.				0.782
Q 11.	Diet is a part of the treatment of diabetes.				0.838
Q 12.	High blood sugar hardly increases the risk of cardiovascular disease.				0.777
	**Total**	**57.041**	**0.887**		

**Table 5:** Differences in the level of knowledge score obtained according to sex and education level.

	Knowledge Level	
	Mean ± SD (CI 95%)	*p*
**Sex**		
Men	105.97 ± 9.54 (0.07–7.76)	0.046
Women	102.96 ± 9.77 (0.07–7.76)	
**Education level**		
Low	99.20 ± 11.93 (93.61–104.79)	0.003
Moderate	105.92 ± 7.85 (103.30–108.54)	
High	109.78 ± 8.76 (105.42–114.13)	

## Data Availability

Not applicable.
